# Fabrication of Periodic Plasmonic Structures Using Interference Lithography and Chalcogenide Photoresist

**DOI:** 10.1186/s11671-015-1203-x

**Published:** 2015-12-29

**Authors:** Viktor Dan’ko, Mykola Dmitruk, Ivan Indutnyi, Sergii Mamykin, Victor Myn’ko, Mariia Lukaniuk, Petro Shepeliavyi, Petro Lytvyn

**Affiliations:** V. Lashkaryov Institute of Semiconductor Physics, National Academy of Sciences of Ukraine, 45, Prospect Nauky, 03028 Kyiv, Ukraine

**Keywords:** Plasmonic structures, Plasmon resonance, Chalcogenide photoresist, Interference lithography, 73.20 Mf, 85.40 Hp, 42.79.Dj

## Abstract

This study reports on the employment of the interference lithography (IL) technique, using photoresist based on the chalcogenide glass (ChG) films, for fabrication of one-dimensional (gratings) and two-dimensional (arrays) periodic plasmonic structures on the surface of glass plates. The IL technique was optimized for patterning of the Au and Al layers and formation of gratings and arrays with a spatial frequency of 2000 mm^−1^. Optical properties of obtained structures were studied using measurements of spectral and angular dependence of transmission and reflection of polarized light. It was shown that the spectral and angular position of the surface plasmon polariton and local surface plasmon resonance, which are observed in these samples, can be adjusted over a wide range by selecting the geometric parameters of structures and technological modes of their manufacturing.

## Background

In recent years the attention of many researchers has attracted by metal plasmonic structures that play an important role in numerous fields of research and applications, for example, as a substrate for optical sensors based on plasmon resonance [1], or surface-enhanced Raman spectroscopy [2] as sub-wavelength optical elements [3], and others. These structures are formed by modern lithographic technologies: electron-beam or ion-beam lithography [4, 5], nanosphere lithography [6], nanoimprint lithography [7], deep ultraviolet lithography [8], and others. But all this techniques are expensive and poorly suitable for large area processing.

At the same time, the more simple and technological are interference lithography (IL), which can be used for the rapid fabrication of wafer-scale periodic nanostructures [9, 10]. IL is a large area fabrication technique that uses laser interference patterns for rapid formation of periodic structures such as gratings and bi-gratings (arrays). The total processed area depends on the beam intensity and coherence length of the laser and can be up to dozens, or even hundreds of square centimeters. For electron and ion processes, the one write-field typically is equal to several hundreds of square micrometers. For nanoimprint lithography based on the replication of nanostructures inscribed in a stamp using the same electron-beam lithography, the fabrication of the stamps is a slow and costly process. In addition, these stamps’ lifetime is no more than 100 prints [11]. And even for nanosphere lithography, due to the non-zero size dispersion of the nanospheres, formed two-dimensional structure is divided into differing domains with sizes less than 100 μm.

In previous studies the authors have shown that IL with the use of chalcogenide photoresist is a promising technology for the formation of one- and two-dimensional submicron periodic structures on the surface of semiconductors and dielectrics [9, 12]. Chalcogenide photoresists based on thermal evaporated amorphous films of chalcogenide glasses (ChG) are characterized by high-resolution, optical uniformity, wide spectral range of photosensitivity [13, 14]. In addition, these photoresists possess a high refractive index ranging from 2.0 to 3.0 and are very perspective for immersion IL [9].

Photostimulated structural changes in vacuum-deposited films of chalcogenide glass have three components: reversible, irreversible, and transient (which occurs only during exposure of the photoresist and rapidly relax after switching off the light) [14, 15]. Using of chalcogenide films as photoresist traditionally was associated exactly with irreversible changes in their solubility. Recently, the possibility of realization IL on the reversible and transient photoinduced structural change of chalcogenide films was shown by authors of works [16, 17].

In this paper, we present investigations of the formation processes of one-dimensional (gratings) and two-dimensional (arrays) periodic metal structures on the surface of glass plates using IL and chalcogenide photoresist. The paper also studies the influence of thermal treatments on morphological characteristics and features of excited plasmons in golden submicron periodic structures.

## Methods

The samples for our experiments were prepared by successive thermal vacuum deposition of 3-nm-thick (effective thickness) Cr adhesive layer, a layer of metal (Au) with a thickness of 20–120 nm and photoresist layer (As_2_S_3_ or GeSe_3_) with thicknesses from 50 to 300 nm onto polished glass substrates with a size of 50 mm × 50 mm at a residual pressure of 2 × 10^−3^ Pa. The deposition rate and films thicknesses were monitored in situ with a KIT-1 quartz thickness meter. After deposition, the film thicknesses were controlled using a MII-4 microinterferometer.

The recording of periodic structures on annealed ChG films was carried out using the interference pattern formed with a helium-cadmium laser (wavelength *λ* = 440 nm). The exposition value for the gratings recording (1D structures) was 0.2–0.5 J/cm^2^, and during the recording of the bi-gratings (2D structures), each exposition was decreased by 1.3–1.5 times. Two-dimensional periodic structures were formed by double exposure with the rotation of the sample between expositions on 90° around the normal. After exposure, the samples were chemically treated in non-water alkaline organic solutions to form a resistive mask in photoresist layer, through which the metal film was etched. After removing the photoresist residues in alkaline solution, washing and drying the metal periodic structure was obtained.

The surface patterns of obtained structures were examined with a Dimension 3000 Scanning Probe atomic force microscope (Digital Instruments Inc., Tonawanda, NY, USA) in the atomic force microscope (AFM) tapping mode.

Optical properties of fabricated 1D and 2D structures were studied using measurements of spectral and angular dependence of transmission and reflection of polarized light in the wavelength range 0.4–1.1 μm and angles of incidence of 10°–80°. The automated setup for such measurements consists of illuminator, a mechanical light chopper, a monochromator with Glan prism at the exit, and rotary table for samples. The intensity of the light reflection or transmission is measured by silicon photodetector; signal of which after amplification and demodulation is applied to the input of analog-to-digital converter. Such measurements allow to build the dispersion curves of excited optical modes and to identify their type.

## Results and Discussion

To create a chalcogenide lithographic mask with specified parameters using IL, you must select the optimal thickness of photoresist, exposure time, selectivity of etchant, and time of the post-exposure etching. IL technology was applied in a mode of little over exposure of photoresist to provide a cycloid form of groove profile of periodic chalcogenide mask. By changing the time of selective etching of photoresist, it is possible to change the width of the elements of lithographic masks and, accordingly, the width of opened intervals between the elements of the mask through which there is a further etching of the metal layer. Etching of the photoresist was monitored in situ by registration of non-photoactive long wavelength light diffracted from relief structure which is formed in photoresist layer. For a given photo-resistive mask, a form of profile and duty cycle (the ratio of width to period) of the elements in the periodic structure, obtained as a result of wet etching of the metal layer through chalcogenide mask, were determined also by rate and time of metal etching.

**Fig. 1:**
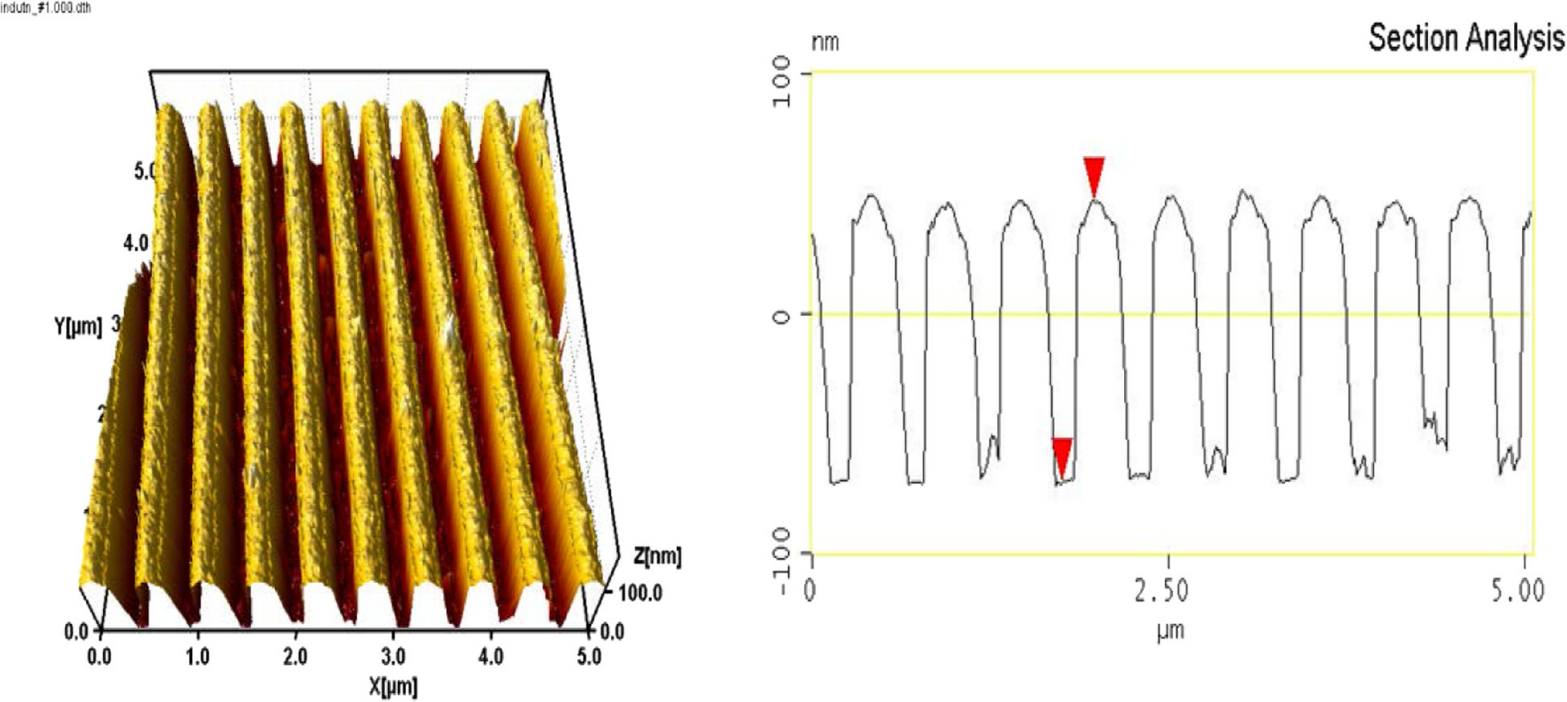
The phase plate based on sub-wavelength Al grating. Half-width of metal groove—280 nm; height—115 nm; groove sectional form is close to trapezoid

**Fig. 2:**
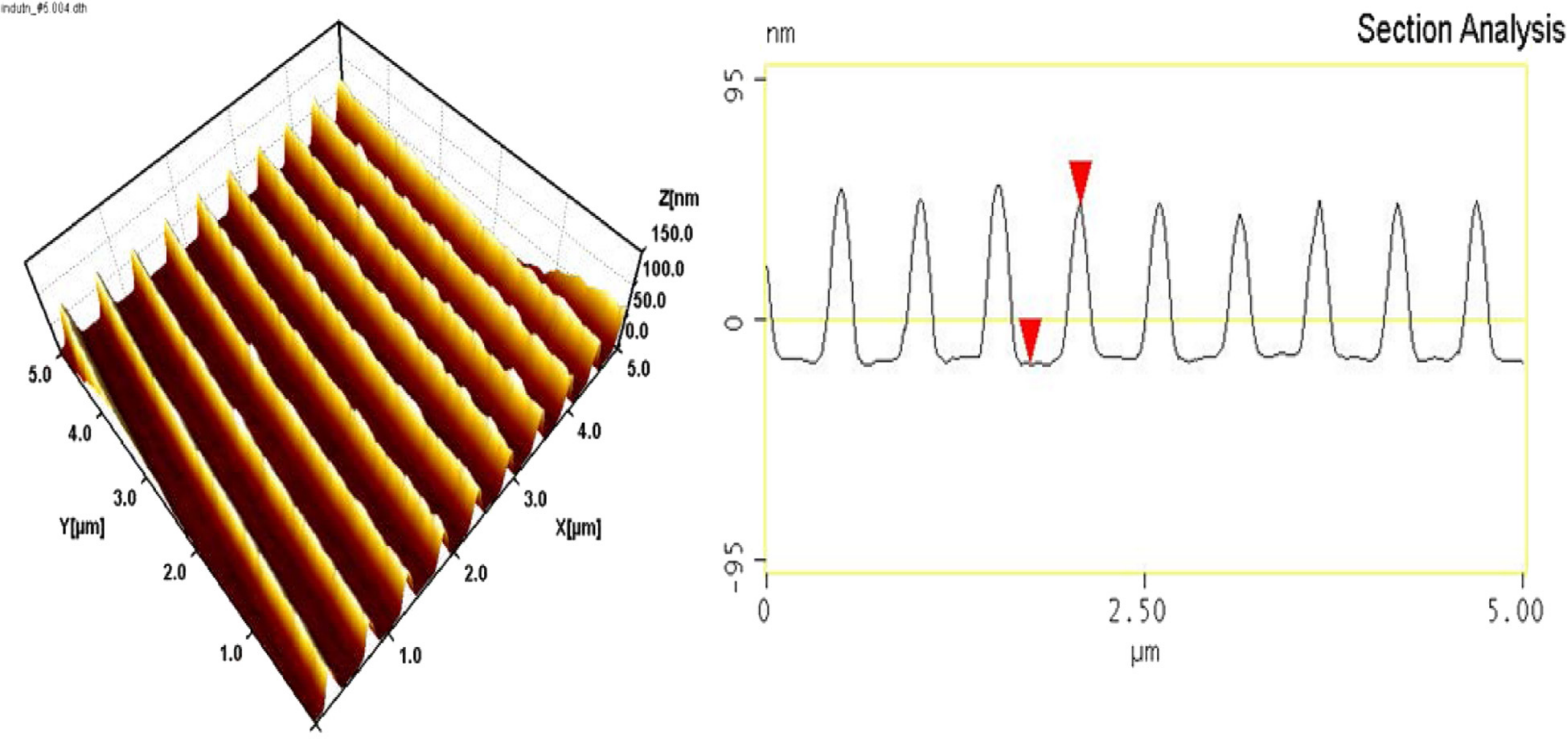
The phase plate based on sub-wavelength Al grating. Half-width of metal groove—130 nm; height—65 nm; groove sectional form is close to triangular

Figures 1 and 2 show AFM images and sectional profile of Al gratings with a period of 500 nm (2000 mm^-1^ spatial frequency) prepared by IL using chalcogenide photoresist. In the process of their production on both samples, the chalcogenide lithographic masks with identical parameters (thickness and duty cycle) were formed, but the etching of the metal layer in the etchant based on hydrochloric acid was different: 20 s for the grating in Fig. 1 and 40 s for the grating in Fig. 2. As seen from the figures, formed sub-wavelength metal structures are substantially different in duty cycle and form of groove section. Increased time of etching leads to a reduction in the width of groove (with significant “over-etching” decreasing groove height), and the form of section is changed from trapezoid to triangular.

Another method for controlling form of elements in periodic metallic structures is thermal treatment (annealing). Most clear effect of annealing manifested in the gold plasmonic structures. In [18] samples of thermally deposited gold layers with an effective thickness from 1 to 12 nm were investigated in detail, in particular, the effect of annealing in air at temperatures from 250 to 450 °C on morphological and optical properties of disordered Au nanoislands. It was found that in the temperature range 350–450 °C, Au nanoislands of the spherical and ellipsoidal shapes are formed, and the morphology of the Au island film and their plasmon resonance spectrum depends noticeably on the nominal Au thickness and post-annealing temperature, while the duration of annealing is of minor importance.

This paper studied the effect of annealing in vacuum at temperatures from 350 to 450 °C on morphological and optical properties of Au periodic structures, formed by IL using chalcogenide photoresist. It was found that the optimum temperature for our samples with Au thicknesses from 30 to 60 nm is 400 °C—at lower temperatures insufficient effect of annealing, at 450 °C begins formation of agglomerates and disordering of the structure.

**Fig. 3:**
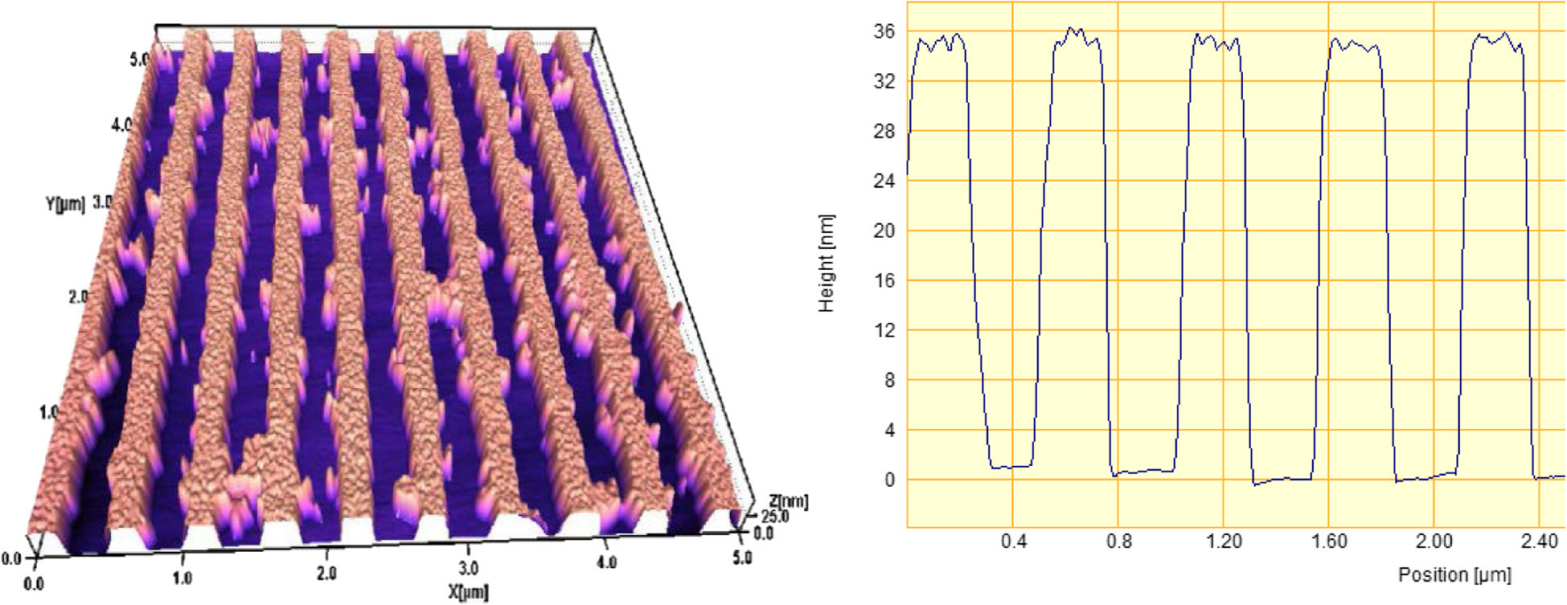
AFM image and cross-sectional profile of the Au grating with period of 500 nm

Figure 3 shows AFM image and cross-sectional profile of the Au grating with period of 500 nm formed by IL on a glass substrate. Duty cycle of the structure is close to 50 %, thickness of the gold is 35 nm, and the groove sectional form is trapezoid, even close to rectangular.

**Fig. 4:**
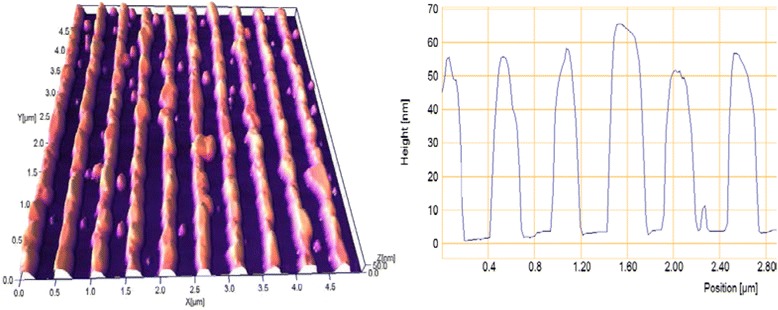
The same grating as in Fig. 3 after annealing at 400 °C for 15 min

This grating was annealed in vacuum at 400 °C for 15 min. Figure 4 shows the image and groove profile of the same grating after annealing. It is seen significant changes in the groove profile, as a result of annealing. Trapezoid flat-topped groves were transformed in convex grooves with smaller half-width and greater height. Investigation of groove heights histogram has shown that the initial height of the grooves of 35 ± 2 nm has increased to 54 ± 10 nm, as a result of the annealing. That is, the flat Au nanostrips were transformed during the annealing into semi-cylindrical nanowires by the forces of surface tension.

**Fig. 5:**
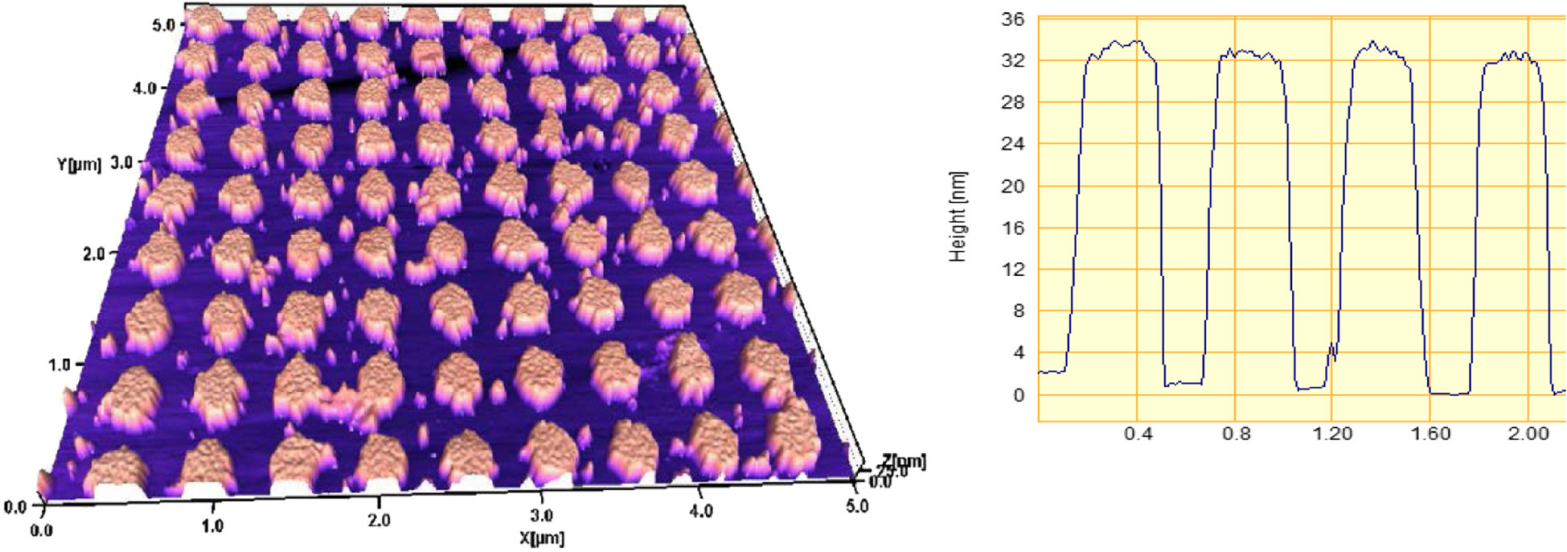
AFM image and cross-sectional profile of laterally ordered Au two-dimensional structure with a period of 500 nm

The influence of thermal annealing on the ordered two-dimensional plasmon structures is significantly greater. Figure 5 shows AFM images and cross-sectional profile of Au bi-grating with the same period of 500 nm formed by IL with a double exposure. The form of elements of this two-dimensional periodic structure is close to the deformed cylinders with flat tops.

**Fig. 6:**
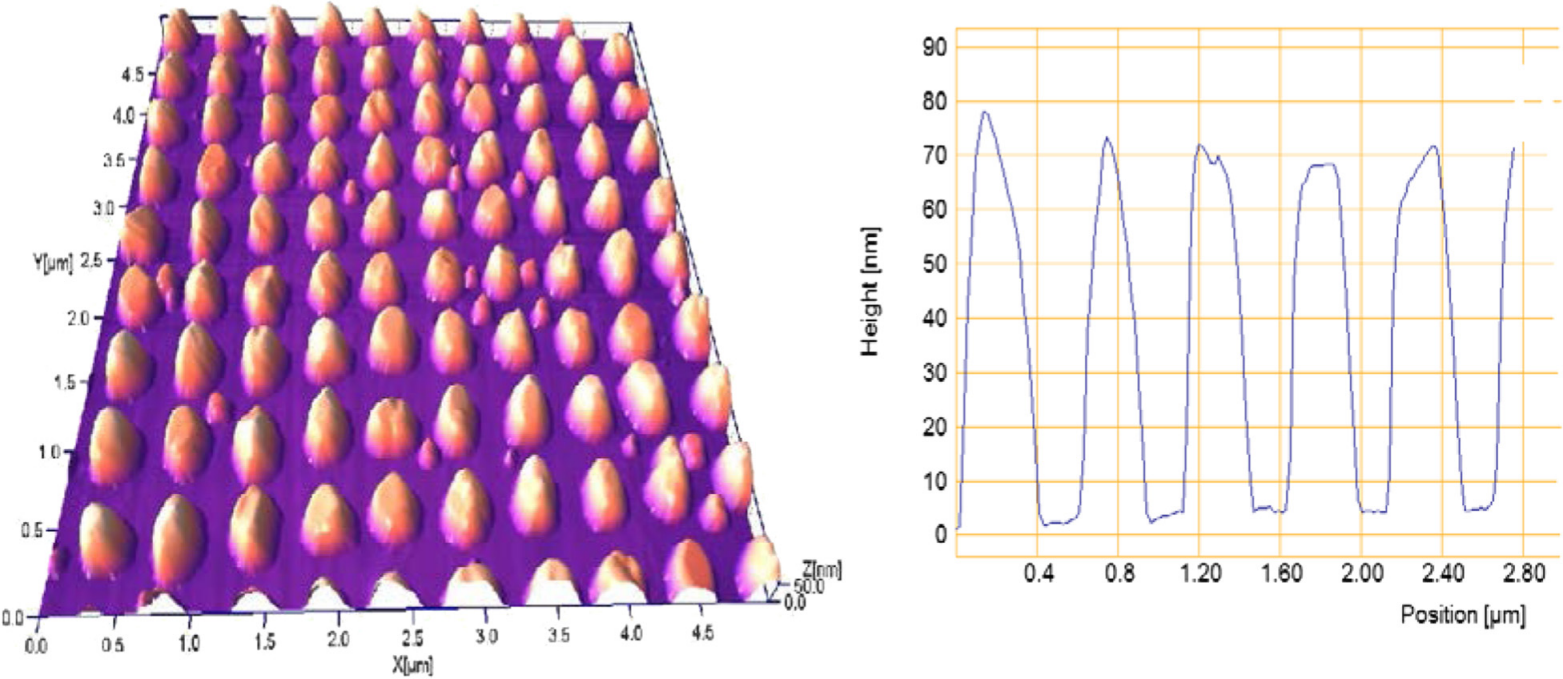
The same two-dimensional structure as in Fig. 5 after annealing at 400 °C for 15 min

Figure 6 illustrates transformation of these elements as a result of annealing in vacuum at 400 °C for 15 min. Cylindrical form of Au island was transformed into dome-shaped with a smaller half-width and much higher altitude. The initial height of the elements of 34 ± 2 nm has increased up to 64 ± 12 nm, as a result of annealing. This is significantly higher than for gratings, as in this case, «shrinkage» of Au island is two-dimensional, in contrast to the grating, where the process is one-dimensional.

**Fig. 7:**
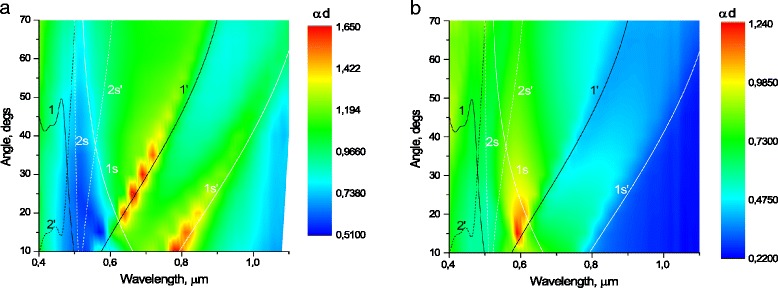
P-polarized extinction as a function of wavelength and angle of incidence, of Au grating with period of 500 nm unannealed (**a**) and annealed at 400 °C for 15 min (**b**) overlapped with the dispersion curves calculated from Eqs. (1) and (2): the dispersion curves of the surface plasmon polaritons corresponding to the air-metal interface (1, 1′, 2′) and substrate-metal interface (1s, 1s′, 2s, 2s′) for *m* = +1 (1, 1s), *m* = +2 (2s), *m* = −1 (1′, 1s′) and *m* = −2 (2s′) diffraction orders. The *color bar* shows extinction with *red* representing high extinction

**Fig. 8:**
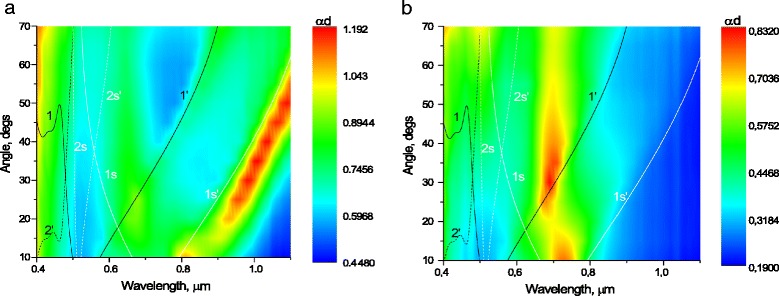
P-polarized extinction as a function of wavelength and angle of incidence, of Au two-dimensional structure with a period of 500 nm unannealed (**a**) and annealed at 400 °C for 15 min (**b**) overlapped with the dispersion curves calculated from Eqs. (1) and (2): the dispersion curves of the surface plasmon polaritons corresponding to the air-metal interface (1, 1′, 2′) and substrate-metal interface (1s, 1s′, 2s, 2s′) for *m* = +1 (1, 1s), *m* = +2 (2s), *m* = −1 (1′, 1 s′) and *m* = −2 (2s′) diffraction orders. The *color bar* shows extinction with *red* representing high extinction

Modification of morphological characteristics of the plasmon structures have displayed in their optical characteristics. The extinction of incident P-polarized light, *αd* = ln((1-*R*)/*T*) (where *R*—reflectance, T—transmittance) is shown in Figs. 7 and 8 for gratings from Figs. 3 and 4 and two-dimensional structures from Figs. 5 and 6, accordingly. Bands of high extinction are the result of a resonant excitation of surface plasmon polaritons (SPPs) and local plasmons (LP), mapping thereby the dispersion curves of the plasmon resonances.

The spectral-angular position of the resonance peak of the incident radiation conversion into surface plasmon polariton waves is determined by equating the components of SPP wave vector and wave vector of the incident radiation, which parallel to the interface between the metal and non-metallic environment [19, 20]:

1 $$ k\  \sin \theta +mG={k}_{\mathrm{PP}}, $$

where *k* = 2*π*/(*λ/n*)—wave vector of the incident radiation with a wavelength *λ* in a vacuum; *θ*—angle of incidence; *m*—an integer (*m* ≠ 0) and denotes the diffraction order; ***G*** *=* 2π*/a*—reciprocal vector of grating with a period of *a*, *ε = n*^2^—permittivity and refractive index of the environment; and *k*_PP_—wave vector of SPP.

To estimate approximately the magnitude of SPP wave vector, the expression, obtained for a flat interface of two semi-infinite media [19], can be applied

2 $$ {k}_{\mathrm{PP}}=\pm \left(2\pi /\left(\lambda /n\right)\right)\ {\left[{\upvarepsilon}_{\mathrm{Me}}\upvarepsilon /\left({\upvarepsilon}_{\mathrm{Me}}+\upvarepsilon \right)\right]}^{1/2}, $$

where *k*_PP_ has “+” sign at m > 0 and “−” at m < 0. Here, *ε*_Me_ = *ε*′_Me_ + *iε*″_Me_ = (*n + ik*)^2^—complex permittivity of the metal at the wavelength *λ*.

By using the expressions (1–2), the dispersion curves for SPPs, excited at interfaces metal/air or metal/substrate, were built in the coordinates “angle of excitation” versus “wavelength,” which is shown in Figs. 7 and 8. For calculations, the optical constants of gold from [21] were used, and the refractive index of the substrate was taken equal to *n*_*s*_ = 1.48. For a given gratings geometry and optical constants, it is possible excitation of modes with *m* = +1 (1, 1s); *m* = +2 (2, 2s); *m* = −1 (1′, 1s′); *m* = −2 (2′, 2s′); at the interface air/gold (1, 1′, 2, 2′) and gold/substrate (1s, 1s′, 2s, 2s′).

The peculiarity of LP excitation in a periodic array of nanowires is a weak dependence of the LP resonance spectral position on the angle of incidence [22], which is mostly due to angular dependence of the width of nanowires projection in the plane of light wave vector *k*_*x*_. To estimate the upper high-frequency spectral position of the LP resonance, one can use the formula for the polarization *α* of the spherical metal nanoparticle in dielectric environment [23]:

3 $$ \alpha =\left({\varepsilon}_{\mathrm{Me}}/\varepsilon -1\right)/\left({\varepsilon}_{\mathrm{Me}}/\varepsilon +2\right), $$

which in the case of gold nanoparticle in a vacuum reaches a maximum in the vicinity of *λ* ~ 0.5 μm. With increasing the nanoparticle size (width of nanowires) and/or dielectric constant of the environment, the resonant wavelength can significantly increase [24] and reach the infrared region of the spectrum; it also increases if in the nearest vicinity other metal nanoparticles are located—due to the interaction between them.

Excited SPP waves with *m* = −1 are most intense on the unannealed samples of diffraction gratings (1′, 1s′ Fig. 7a), because the edges of grating grooves (nanowires) are closer to each other, than in annealed structures, and SPP meets with less resistance when spread along the surface. Due to considerable contact area between nanowires and substrate for the unannealed samples, the mode, which excited on the gold-substrate interface (1s′ on Fig. 7a), is intense. Excitation of LP is hardly noticeable on the background of intense SPPs.

After annealing, due to «shrinkage» of Au nanowires and islands in a more compact structure and, consequently, increase the distance between the edges of nanowires or nanodots, the intensity of SPP decreases, especially decreases mode 1s′, while mode 1′ (excited on the gold/air interface) remains moderately intense (Fig. 7b). Also, after annealing on the background of a significant reduction of SPP intensity, the LP intensity increases significantly, which is most clearly seen on the annealed 2D structure (Fig. 8b) in spectral region *λ =* 0.6–0.8 μm.

## Conclusions

It is shown that IL technology using vacuum chalcogenide photoresist in combination with additional thermal treatment enabled the successful fabrication of uniform plasmonic structures with required characteristics (spatial frequency, depth of relief, a form of element profile) on a large area substrates (up to 25 cm^2^). The results of optical measurement confirm the excitation of surface plasmon polariton and local surface plasmon resonance in fabricated samples, spectral, and angular position of which coincides with the predictions of the theory and can be adjusted over a wide range by selecting the geometric parameters of structures and technological modes of their manufacturing.

## Abbreviations

AFM: atomic force microscope
ChG: chalcogenide glasses
IL: interference lithography
LP: local plasmons
SPPs: surface plasmon polaritons
